# Effect of a community health worker mHealth monitoring system on uptake of maternal and newborn health services in Rwanda

**DOI:** 10.1186/s41256-019-0098-y

**Published:** 2019-03-22

**Authors:** Celestin Hategeka, Hinda Ruton, Michael R. Law

**Affiliations:** 1 Centre for Health Services and Policy Research, Faculty of Medicine, School of Population and Public Health, The University of British Columbia, 201-2206 East Mall, Vancouver, BC V6T1Z3 Canada; 2 Collaboration for Outcomes Research and Evaluation, Faculty of Pharmaceutical Sciences, The University of British Columbia, Vancouver, BC Canada; 3 School of Public Health, College of Medicine and Health Sciences, University of Rwanda, Kigali, Rwanda

**Keywords:** Maternal and newborn health, mHealth, RapidSMS, Interrupted time series analysis, Rwanda demographic and health survey

## Abstract

**Background:**

In an effort to improve access to proven maternal and newborn health interventions, Rwanda implemented a mobile phone (mHealth) monitoring system called RapidSMS. RapidSMS was scaled up across Rwanda in 2013. The objective of this study was to evaluate the impact of RapidSMS on the utilization of maternal and newborn health services in Rwanda.

**Methods:**

Using data from the 2014/15 Rwanda demographic and health survey, we identified a cohort of women aged 15–49 years who had a live birth that occurred between 2010 and 2014. Using interrupted time series design, we estimated the impact of RapidSMS on uptake of maternal and newborn health services including antenatal care (ANC), health facility delivery and vaccination coverage.

**Results:**

Overall, the coverage rate at baseline for ANC (at least one visit), health facility delivery and vaccination was very high (> 90%). The baseline rate was 50.30% for first ANC visit during the first trimester and 40.57% for at least four ANC visits. We found no evidence that implementing RapidSMS was associated with an immediate increase in ANC (level change: -1.00% (95% CI: -2.30 to 0.29) for ANC visit at least once, -1.69% (95% CI: -9.94 to 6.55) for ANC (at least 4 visits), -3.80% (95% CI: -13.66 to 6.05) for first ANC visit during the first trimester), health facility delivery (level change: -1.79, 95% CI: -6.16 to 2.58), and vaccination coverage (level change: 0.58% (95%CI: -0.38 to 1.55) for BCG, -0.75% (95% CI: -6.18 to 4.67) for polio 0). Moreover, there was no significant trend change across the outcomes studied.

**Conclusion:**

Based on survey data, the implementation of RapidSMS did not appear to increase uptake of the maternal and newborn health services we studied in Rwanda. In most instances, this was because the existing level of the indicators we studied was very high (ceiling effect), leaving little room for potential improvement. RapidSMS may work in contexts where improvement remains to be made, but not for indicators that are already very high. As such, further research is required to understand why RapidSMS had no impact on indicators where there was enough room for improvement.

## Introduction

Improving maternal, newborn and child health outcomes remains one of the most significant challenges for low- and middle- income countries (LMICs) [1, 2]. Reducing under-five child and maternal mortality by two-thirds and three-quarters between 1990 and 2015 were two of the eight United Nations Millennium Development Goals (MDG) [3]. Globally, considerable improvement in maternal, newborn, and child survival has been registered over the MDG era, with the maternal mortality ratio (MMR) reduced by 45% and under-five mortality reduced by more than half (53%) by 2015 [4]. Nevertheless, the improvement has not been homogenous across countries and, in fact, only a third of countries (62) achieved the fourth MDG while only nine countries met the fifth MDG by 2015 [1, 2].

Rwanda has made impressive progress with regards to improving maternal and newborn and child survival post-1994 genocide [5–7]. Although Rwanda achieved the maternal and child health-related MDGs, the neonatal mortality rate (MNR) and maternal mortality ratio (MMR) remain high and could be reduced significantly with timely access to quality health services across the continuum of care for mothers and newborns [8]. Rwanda’s NMR and MMR are currently estimated at 20 deaths per 1000 live births and 210 deaths per 100,000 live births, respectively [9]. Therefore, efforts are still required for the country to achieve the recently adopted United Nations’ Sustainable Development Goals (SDGs) (i.e. SDG targets # 3.1 and 3.2: reduce maternal mortality to less than 70 per 100,000 live births and neonatal mortality to at least as low as 12 per 1,000 live births by 2030) [10]. Currently, the leading causes of neonatal mortality include birth asphyxia, prematurity, and neonatal infections; while hemorrhage, obstructed labor, sepsis, and hypertensive disorders of pregnancy are the leading causes of maternal deaths in Rwanda [11, 12]. Most of these deaths are preventable with timely access to proven maternal and newborn health interventions [1, 13]. Unfortunately, while the knowledge of most of the interventions needed to improve maternal and newborn survival has advanced remarkably since the adoption of the MDGs, healthcare systems in many LMICs do not effectively deliver currently recommended life-saving maternal and newborn health interventions including antenatal and post-natal care [1, 13].

Evidence suggests that effective coverage of proven health interventions across the continuum of maternal and newborn care could lead to a substantial reduction in maternal and neonatal mortality in LMICs [8, 10]. Bhutta and colleagues estimated that increasing coverage and quality of preconception, antenatal, intrapartum and postnatal interventions (already proven to be effective) by 2025 could prevent approximately two-thirds of neonatal deaths, one-third of stillbirths, and over half of maternal deaths annually [8]. In an effort to increase uptake of proven maternal and newborn healthcare services and, ultimately, improve maternal and newborn health, Rwanda has been using an SMS based alert system called RapidSMS [14]. RapidSMS is a free and open source platform for mobile alert systems [15]. This mhealth platform was modified for use in Rwanda to facilitate communication between community health workers and the ambulance system, health facilities staff, and the central government.

The use of mhealth interventions has been increasing globally, with approximately 83% of World Health Organization (WHO) member countries reporting having one mhealth initiative in 2016 [16]. At the same time, however, there is a paucity of evaluations undertaken in these countries to provide evidence on whether these mhealth programs are indeed effective [16]. Therefore, the objective of this study was to evaluate the impact of the RapidSMS program on the utilization of maternal and newborn healthcare services in Rwanda.

## Methods

### Study context

Rwanda is a small, landlocked, and low-income country located in East Africa, with a population of over 11 million [17]. Community health workers (CHWs), who follow up pairs of mother-infant in their villages, were trained and equipped with mobile phones to allow interactive real time two-way communication with the health ambulance system (in case of emergency), health facility staff, district hospital and the central level for action to improve access to antenatal care, postnatal care, health facility delivery, and emergency obstetrical and neonatal care [14]. These CHWs identify pregnant women in their village and make follow-ups across the continuum of care for maternal, newborn and child health to improve utilization of recommended health services [14].

Using mobile phones provided as part of the RapidSMS program, CHWs record new pregnancies (including mother/infant identification and the first day of the last menstrual period) into the RapidSMS system. They also collect danger signs and symptoms suggestive of potential life-threatening event that warrant urgent attention [14]. RapidSMS tracks mothers and infants through two years of age. Based on pregnancy-specific information, the RapidSMS system generates and sends SMS reminders to CHWs, along with the pairs (mother/infant) they are tasked to follow up, dates for clinical appointments including antenatal visits, delivery and postnatal care visits [14]. By tracking pregnancies and providing reminders, RapidSMS could potentially increase utilization of recommended maternal and newborn healthcare services including antenatal care visits, health facility deliveries, receipt of postnatal care, and immunizations.

While maternal newborn and child health has considerably improved across Rwanda over the past several years, 10/30 districts lagged behind and, therefore, benefited from additional support from UNICEF including CHWs training and quarterly supervision meetings [18]. When CHWs report danger signs, the RapidSMS system generates an emergency alert system (RED alert notification system) comprising of “immediate feedback to the CHW advising on immediate action” [14]. Furthermore, it sends an ambulance request to the closest ambulance vehicle point [14]. Rwanda is divided into 30 districts and the RapidSMS program was implemented across these districts at different time points. While 29 districts scaled up the program at different points in 2013, one district (Musanze) did so in 2010 [14] and was therefore excluded from our evaluation.

### Data source

We investigated the impact of the implementation of RapidSMS on uptake of maternal and newborn healthcare services including antenatal care visits, health facility deliveries, and immunizations. Our data came from the Rwanda Demographic and Health Survey (RDHS) [9]. The RDHS is a population-based cross-sectional survey carried out every five years by the National Institute of Statistics of Rwanda in collaboration with the DHS Program ICF International using complex multi-stage sampling to gather a nationally representative sample [9]. Through face-to-face interviews, this survey collects data including on health services utilization across the continuum of care for maternal, newborn and child health [9]. The most recent wave of RDHS (2014/15 RDHS) was carried out from November 2014 to April 2015 and is a nationally representative survey of 12,699 households, 13,497 women aged 15–49 years and 6217 men aged 15–59 years [9]. The 2014/15 RDHS collected data covering five years preceding the survey (2010–2014) [9].

For this program analysis, we analyzed aggregated data from 24 months prior to RapidSMS implementation and 18 months following the implementation of the RapidSMS program across the 29 districts. This length of study time provided adequate data points to construct our longitudinal models [19], as described in the statistical analysis sub-section below.

### Study cohort

Using the RDHS, we identified a cohort of women aged 15–49 years who had a live birth within five years preceding the survey. Using the date of childbirth, we selected births that occurred in the study time and also extracted self-reported information pertaining to healthcare utilization by mothers and newborns, including ANC visits, place of delivery and vaccination coverage. Our unity of observation is the person-month.

### Outcome measures

We studied the impact of RapidSMS on 6 outcomes within the following 3 categories:

- *Antenatal care:* We calculated the number of ANC (at least one visit), the number of ANC (at least four visits) and the number of timely ANC (first ANC in the first trimester) per 100 live births in our cohort.
- *Health facility delivery:* We calculated the number of delivery at a health facility (also used as a proxy for skilled birth attendants) per 100 live births;
- *Vaccination:* We calculated the number of newborns who received BCG vaccine for prevention of tuberculosis and those who received Polio 0 vaccine for prevention of poliomyelitis infection per 100 live births. These two vaccines are part of Rwandan routine immunization program and are given to newborns at birth [20].

### Design and statistical analysis

Using interrupted time series (ITS) design—one of the strongest quasi-experimental designs [19]—we assessed the impact of the RapidSMS program on uptake of maternal and newborn healthcare services including antenatal care visits, health facility deliveries, and vaccinations. Interrupted time series analysis has widely been used in program and policy evaluation in healthcare [19]. To fit our statistical models, we first determined the proportion for each outcome across all person-months in our dataset as described earlier. Using these aggregate proportions, we fitted segmented regression models for each outcome of interest to estimate changes in level and trend associated with the implementation of the RapidSMS program. Our ITS models took the following general form to model each outcome of interest for program status *j*, at time *t*:

$$ {Outcome}_{jt}={\beta}_0+{\beta}_1\bullet {time}_t+{\beta}_2\bullet {level}_j+{\beta}_3\bullet {trend}_{jt}+{\varepsilon}_{jt} $$

Where *outcome* represents each outcome measure of interest as described earlier (e.g. ANC visit at least once during pregnancy, health facility delivery, vaccination); *time* is the month in study time at time *t* (i.e. 1, 2, 3, 4…); *level* represents whether the RapidSMS program was in place in month; and *trend* represents the month *t* it was after the program implementation. In each ITS model, *β*_*0*_ estimated the baseline uptake of maternal and newborn health services evaluated; *β*_*1*_ estimated monthly trend in the uptake in the period before RapidSMS. For this program analysis, the two coefficients of interest are *β*_*2*_, which indicates any immediate change in the level of each of our outcome of interest after the implementation of RapidSMS, and *β*_*3*_, which indicates any change in the monthly trend in the outcome after the implementation of RapidSMS compared to the trend before RapidSMS. Statistically significant values for these coefficients (*β*_*2*_ and/or *β*_*3*_) would indicate that the RapidSMS program had an impact on uptake of maternal and newborn health services analyzed. *ε*_*jt*_ is the error term representing the variability not explained by the model. As monthly observations may have been correlated over time, we assessed for autocorrelation using Durbin-Watson test and visual plots (autocorrelation function and partial autocorrelation function) and controlled for autocorrelation using appropriate adjustments in a generalized least squares model [19]. Given the RapidSMS program was scaled up across the 29 districts at different times in 2013, we conducted all analyses using study time (the number of months relative to each district’s month of RapidSMS implementation) to standardize each district to the others based their individual start date. All analyses were weighted to account for the complex multi-stage sampling design of RHDS. All analyses were performed using R version 3.4.4 [21].

## Results

### Descriptive characteristics

The descriptive characteristics and responses regarding health services use are shown in Table 1. Nearly all respondents had at least one ANC visit during their most recent pregnancy and had their newborns vaccinated for tuberculosis (BCG). Unadjusted rates for early antenatal care (first ANC visit in the first trimester) and delivery at a health facility were highest among younger women, those with post-secondary education, those who were married or in union, and those in the highest wealth quintile.

**Table 1:** Sociodemographic characteristics and health services use among respondents to the Rwanda Demographic and Health Survey, November 2014–April 2015^a^

	Overall sample (*N* = 7766)	Antenatal care (at least 1 visit)	Antenatal care (at least 4 visits)	Antenatal care (in the 1st trimester)	Health facility delivery	BCG vaccination	Polio 0 vaccination
	*n* (%)	%	%	%	%	%	%
Maternal age							
15–29 years	3802 (49.0)	99.07	45.78	61.69	94.01	98.29	90.57
30–49 years	3964 (51.0)	99.23	41.95	51.74	87.27	98.17	90.46
Education							
no education	1156 (14.9)	98.28	36.96	48.65	81.92	97.04	85.15
primary	5627 (72.5)	99.29	44.07	56.35	91.23	98.24	90.73
secondary	824 (10.6)	99.23	45.25	63.14	96.74	99.71	94.95
post-secondary	158 (2.0)	99.76	74.17	82.85	98.17	98.54	97.28
Place of residence							
urban	1272 (16.4)	98.78	43.90	56.48	96.94	98.25	94.77
rural	6494 (83.6)	99.22	43.80	56.62	89.32	98.22	89.67
Married							
no	1388 (17.9)	98.38	37.08	51.54	89.98	97.72	89.29
yes	6378 (82.1)	99.35	45.54	57.87	90.7	98.34	90.78
Wealth quintile							
lowest	1891 (24.4)	98.73	41.35	54.61	83.99	97.76	86.74
second	1676 (21.6)	99.24	43.55	55.00	90.96	98.03	88.80
middle	1530 (19.7)	99.21	45.17	57.93	90.64	98.46	92.24
fourth	1340 (17.3)	99.7	45.49	57.99	92.77	98.19	91.13
highest	1327 (17.1)	98.99	44.3	58.32	97.16	98.89	95.28

^a^ All estimates are weighted to account for the Rwanda demographic and health survey design. Our study sample included data from 29/30 Rwandan districts which scaled up RapidSMS in 2013

### Effect on antenatal care (ANC) visit

**Fig. 1:**
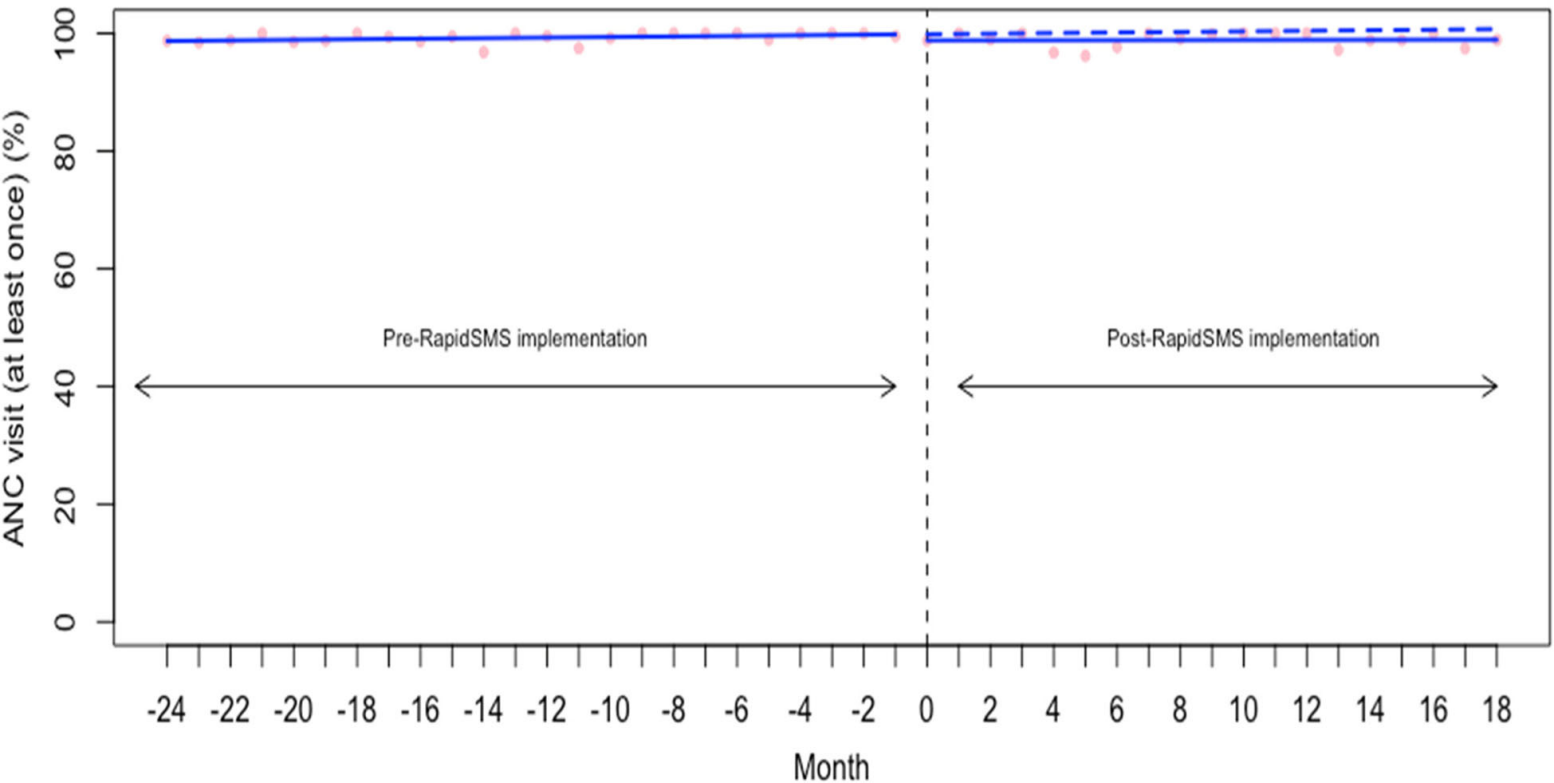
Interrupted time series analysis of antenatal care (at least one visit) during pregnancy

We examined the rate (%) of women with a live birth who had had at least one ANC visit during their most recent pregnancy. The baseline rate of ANC visit at least once during pregnancy was very high at 98.66% (95% confidence interval [CI]: 97.77 to 99.55) (Table 2). As indicated in the Fig. 1 and Table 2, we unsurprisingly found no significant changes in either the level or trend of the receipt of at least one ANC visit during pregnancy (level change: -1.00, 95% CI: -2.30 to 0.29 and trend: -0.04, 95% CI: -0.14 to 0.06). Given the very high level of reported ANC attendance prior to RapidSMS, there was little improvement left to be made on this indicator.

**Table 2:** Baseline coverage rate and change in level and trend for each outcome analyzed

Outcome measures	Baseline coverage rate (95% CI)	Pre- RapidSMS trend (95% CI)	*P* value	Level change (95% CI)	*P* value	Post-RapidSMS trend (95% CI)	*P* value
Antenatal care (ANC)							
ANC (at least one visit)	98.66 (97.77 to 99.55)	0.04 (−0.01 to 0.10)	0.1	-1.00 (−2.30 to 0.29)	0.1	-0.04 (−0.14 to 0.06)	0.4
ANC (at least four visits)	40.57 (34.96 to 46.17)	0.29 (−0.09 to 0.68)	0.1	-1.69 (−9.94 to 6.55)	0.6	-0.40 (−1.09, 0.27)	0.2
ANC visit in the first trimester	50.30 (43.60 to 57.00)	0.52 (0.05 to 0.99)	0.02	-3.80 (−13.66 to 6.05)	0.4	-0.62 (−1.43 to 0.19)	0.1
Childbirth							
Health facility delivery	90.25 (87.27 to 93.22)	0.11 (−0.09 to 0.32)	0.2	-1.79 (−6.16 to 2.58)	0.4	-0.13 (−0.49 to 0.22)	0.4
Immunization							
BCG	98.57 (97.92 to 99.21)	−0.01 (− 0.05 to 0.03)	0.5	0.58 (− 0.38 to 1.55)	0.2	−0.01 (− 0.09 to 0.06)	0.6
Polio 0	91.35 (87.66 to 95.04)	−0.01 (− 0.27 to 0.23)	0.8	− 0.75 (− 6.18 to 4.67)	0.7	0.13 (− 0.31 to 0.58)	0.5

**Fig. 2:**
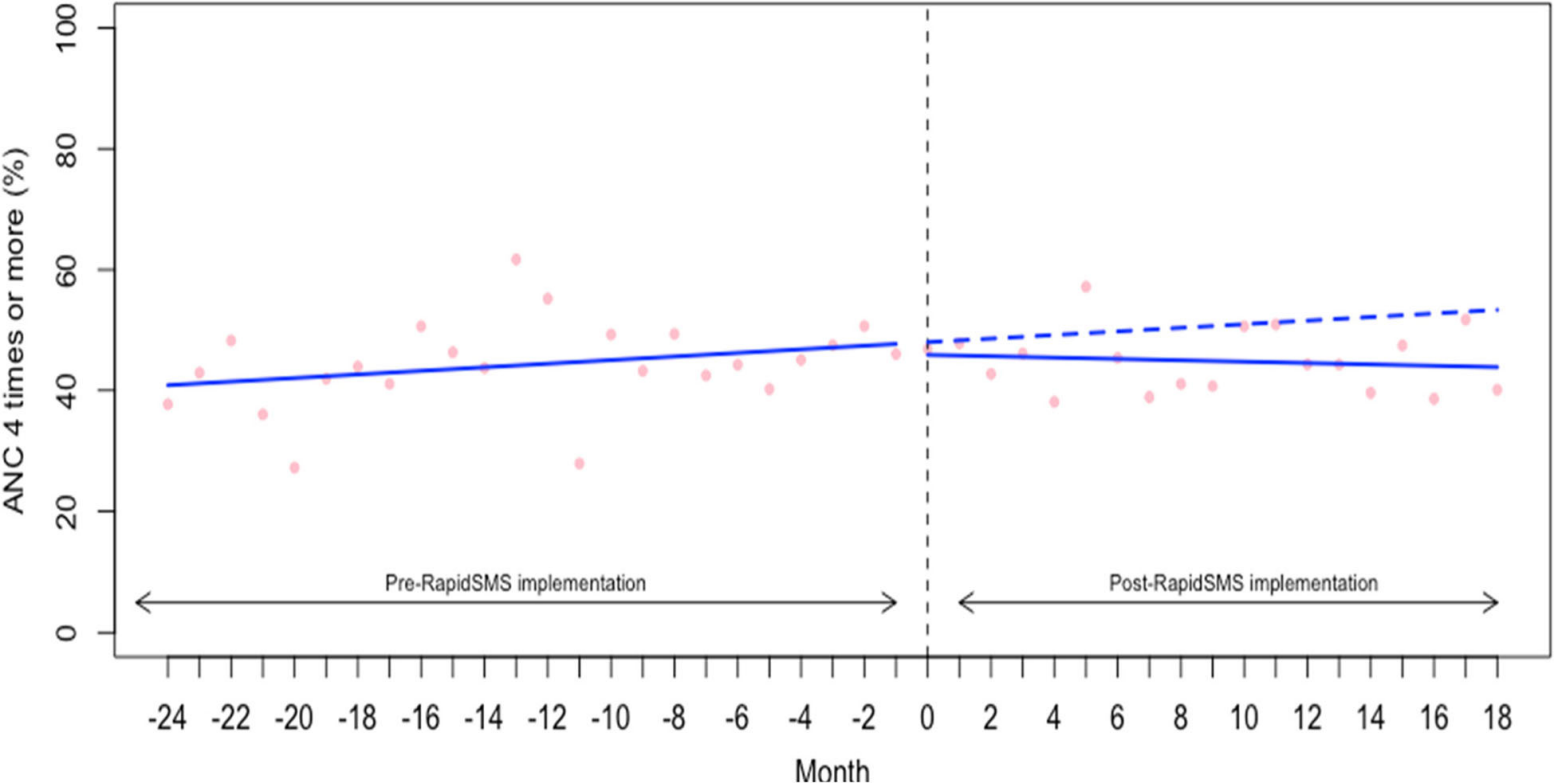
Interrupted time series analysis of antenatal care (at least four visits) during pregnancy

**Fig. 3:**
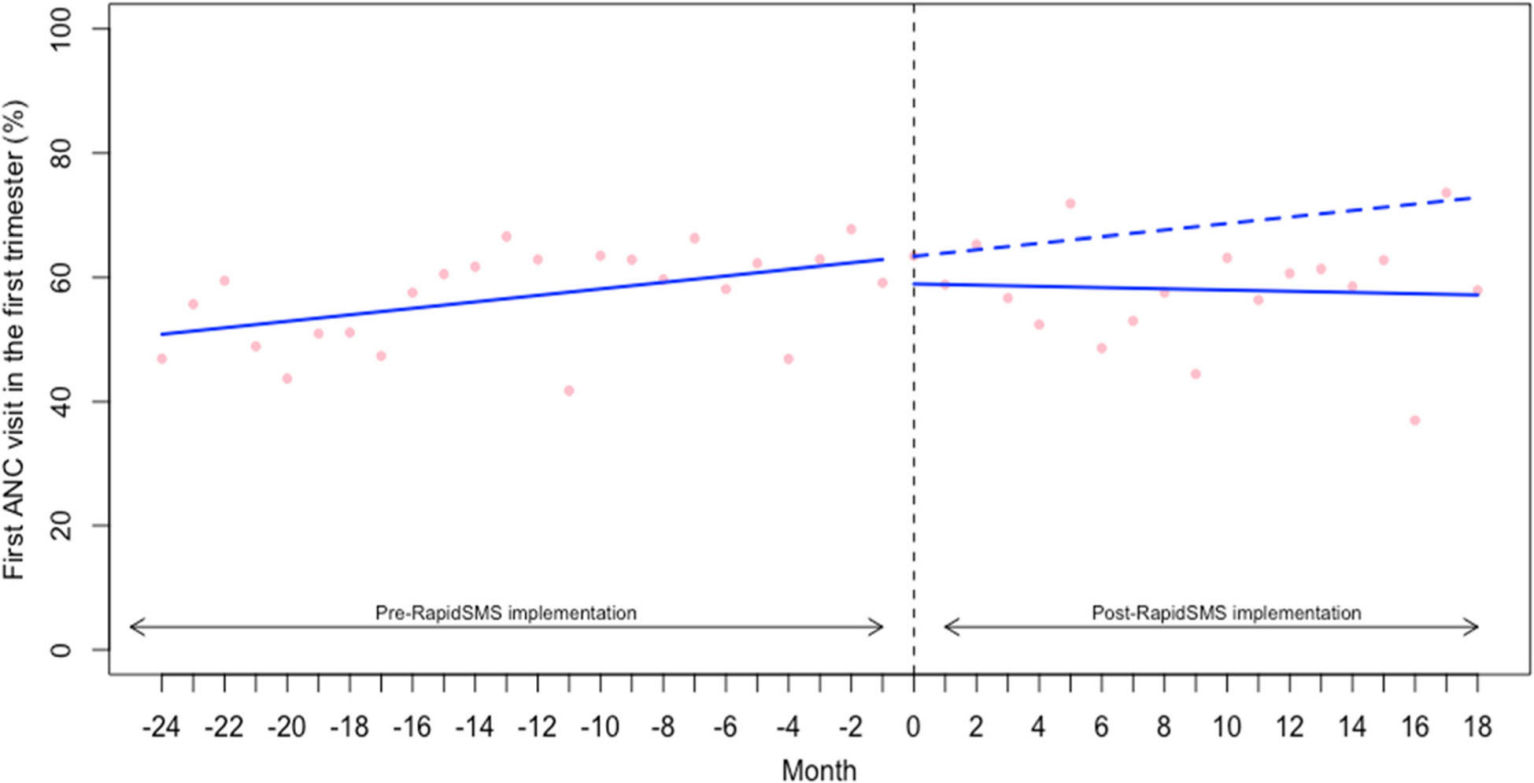
Interrupted time series analysis of antenatal care (first visit in the first trimester)

We also examined the rate (%) of women who completed the recommended standard regimen of 4 ANC visits (or more) during their most recent pregnancy. The baseline rate of 4+ ANC visits was 40.57% (95% CI: 34.96 to 46.17) (Table 2). As shown in the Fig. 2 and Table 2, we did not find that the start of RapidSMS was associated with an improvement in level and trend in 4+ ANC visits (level change: -1.69, 95% CI: -9.94 to 6.55 and trend: -0.40, 95% CI: -1.09 to 0.27). Similarly, we examined the rate of women who had the first ANC visit in the first trimester of the pregnancy (Table 2 and Fig. 3). We did not find evidence that the implementation of RapidSMS was associated with an improvement in level and trend in first ANC visit during the first trimester of the pregnancy (level change: -3.80, 95% CI: -13.66 to 6.05 and trend: -0.62, 95% CI: -1.43 to 0.19) (Table 2 and Fig. 3).

### Effect on health facility delivery

**Fig. 4:**
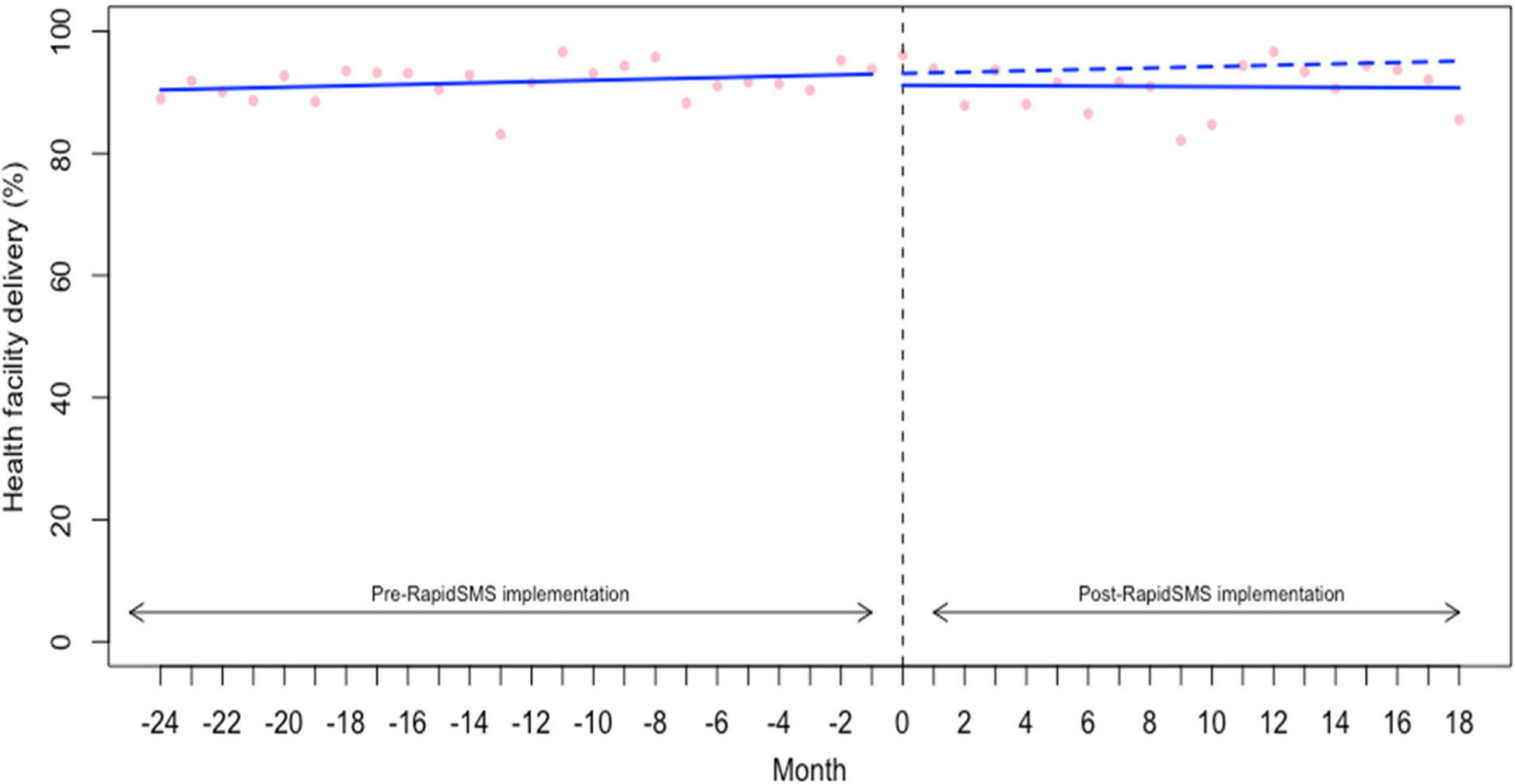
Interrupted time series analysis of health facility delivery

We examined the rate (%) of women with a live birth who delivered at a health facility for their most recent childbirth. As with 1+ ANC visit, the baseline rate of health facility delivery was quite high at 90.25 (95% CI: 87.27 to 93.22) (Table 2). As shown in the Fig. 4, we found no significant changes in either the level or trend in delivery at a health facility (level change: -1.79, 95% CI: -6.16 to 2.58 and trend: -0.13, 95% CI: -0.49 to 0.22).

### Effect on newborn vaccination

**Fig. 5:**
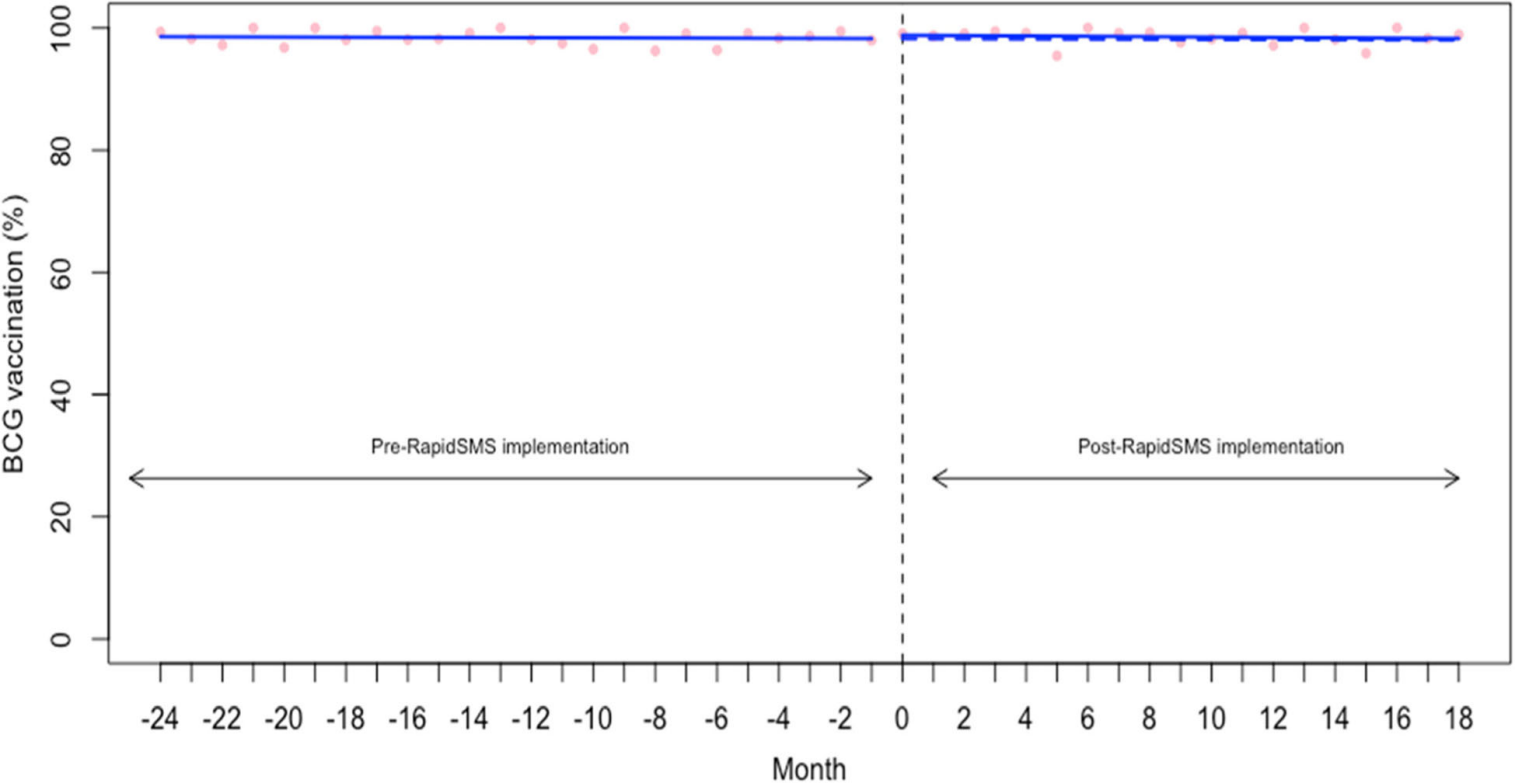
Interrupted time series analysis of BCG receipt for tuberculosis vaccination

**Fig. 6:**
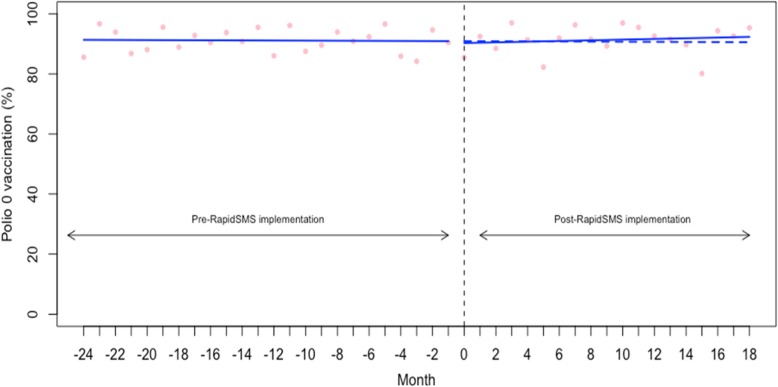
Interrupted time series analysis of polio 0 vaccination

We examined the vaccination coverage rate (%) of BCG (for tuberculosis) and Polio 0 for newborns whose birth occurred in the study time. The baseline coverage rate was very high for both BCG and Polio 0 (98.57% (95% CI: 97.92 to 99.21) and 91.35% (95% CI: 87.66 to 95.04), respectively) (Table 2). As shown in the Figs. 5, 6 and Table 2, we found no significant changes in either the level or trend in BCG and Polio 0 vaccination coverage.

## Discussion

The current evaluative research was undertaken to assess the impact of the RapidSMS program on utilization of maternal and newborn health services including antenatal care visits, institutional deliveries, and vaccination coverage (BCG and polio 0) in Rwanda. Based on our analysis using the most recent wave of the Rwanda Demographic and Health Survey, it appears that the implementation of RapidSMS program did not increase uptake (immediately and over time) of the maternal and newborn health services we studied. In most instances, this was because the existing level of the indicators we studied was very high (ceiling effect), leaving little room for potential improvement.

With increasing access to mobile phone worldwide, implementation of mhealth interventions have been advocated as one of the approaches to improve population health and, approximately 83% of WHO member countries reported having at least one mhealth initiative in 2016 [16, 22, 23]. While hundreds of mhealth interventions have been piloted, little is known about their potential effectiveness at a large scale [16, 22]. During the pilot stage, RapidSMS was found to be associated with increase in the proportion of deliveries occurring in health facilities in Musanze district [14]; however, we found no evidence that the same indicator improved following the nationwide scale-up period. This highlights the importance of evaluating programs regularly even those that were found to be effective at the pilot stage in order to identify potential factors (facilitators and barriers) to their successful adoption and scale-up over time. Nevertheless, RapidSMS may work in contexts where improvement remains to be made, but not for indicators that are already very high.

The existing rates of the uptake for ANC visits (at least once), institutional deliveries and vaccination coverage were very high at over 90%, leaving little room for potential improvement. However, the rate of women who completed the recommended standard regimen of 4 ANC visits (or more) during their most recent pregnancy (40.57%) and the rate of women who had the first ANC visit in the trimester (50.30%) were suboptimal and they did not appear to have increased following RapidSMS implementation. Although further research would be required to understand why RapidSMS had no impact on these indicators where there was enough room for improvement, we believe that the lack of impact could be explained, on the one hand, by the fact that the RapidSMS program was not implemented with perfect fidelity. For example, Musabyimana et al. (2018) qualitative study undertaken in Rwanda to gather the experiences of the RapidSMS program implementers as well as community health workers with implementing RapidSMS identified challenges to successful implementation of RapidSMS including lack of motivation and high turn-over of CHWs, lack of regular CHW training and incentives [24]. Similarly, Condo et al. (2014) identified several other factors (e.g., overwhelming workload and lack of supervision) that could hamper the effectiveness of the Rwandan community health program [25]. This highlights the importance of context and the reality that interventions are rarely implemented with perfect fidelity [26–28].

Going forward, effective RapidSMS implementation strategies should be explored as well as targeting meaningful areas for improvements to be made. For example, given the high penetrance of mobile phones in Rwanda, one may consider using patient-centered SMS reminder as opposite to sending reminders to CHWs. Also, addressing implementation challenges including training and incentivizing CHWs may be helpful [24]. On the other hand, factors beyond the RapidSMS program could also be the reason behind the lack of impact on the indicators where there still was enough room for improvement. For example, growing evidence suggests that women who are not satisfied by the quality of care, including disrespect, they get during their first ANC visits may not come back to health facility for additional ANC visits in order to complete the recommended standard regimen of 4 ANC visits [29]. Growing evidence suggests that quality of health care provided in Rwandan health facilities, like in many other LMICs, is not optimal [30–34]. Delaying in seeking antenatal care (not having first ANC during the first trimester of the pregnancy) has been linked to several factors including knowledge gap, insufficient male involvement in ANC, family income, and maternal age [35–37]. These factors may not be affected by an mhealth intervention such as RapidSMS.

Our findings are consistent with the findings from a concurrent evaluation of the RapidSMS program in Rwanda using routinely collected data from all health facilities across Rwanda (Rwanda Health Management Information System (RHMIS)) and data in the RapidSMS database captured by the community health workers (CHWs) tasked to follow up pairs of mother-infant in their villages in Rwanda [18]. Based on analysis of RHMIS and RapidSMS databases, it was found that the start of RapidSMS had no significant impact on the proportion of women receiving antenatal care in Rwanda [18]. Moreover, the proportion of women delivering at the health facility and that of newborn and mothers receiving postnatal care did not appear to have increased following the implementation of RapidSMS across 20 districts that did not benefit from further CHW training and quarterly supervision [18]. It should be noted that the same indicators improved in the 10 districts that received additional support described earlier [18]. With the size of our sample, we were unable to perform a stratified analysis to formally test whether the impact of RapidSMS differed by the additional support received. Therefore, any impact in the smaller number of supported districts may have been washed out in our national data.

Previous research on the impact of mhealth interventions in other LMICs found increasing evidence around effectiveness of mhealth spanning patient adherence to HIV and tuberculosis treatment and, also healthcare workers’ adherence to treatment guidelines [38–40]. Similarly, in their recent systematic review (*n* = 17 mhealth studies included), Lee and colleagues (2016) looked at the effectiveness of mhealth interventions for maternal, newborn and child health in developing world and found evidence that mhealth interventions using SMS increased the rates of initial breastfeeding and exclusive breastfeeding [41]. However, they found no conclusive evidence on the impact of mhealth interventions on utilization of healthcare services (e.g., ANC, health facility delivery, and vaccination) and on maternal, neonatal and child mortality [41]. It is worthy noting that most of the mhealth studies included in Lee et al’s review were of poor methodological quality [41]. Flaws at the design/implementation stage make it difficult for evaluators to conduct rigorous evaluation. It is advisable for mhealth program implementers to work hand in glove with evaluators to ensure the way mhealth programs are implemented offer opportunity for rigous evaluation. It is also important to evaluate the real-world experiences with implementation of mhealth interventions in order to understand why mheath interventions appear to be effective only in some contexts.

This study has several strengths including use of a nationally representative sample and use of one of the strongest quasi-experimental designs (ITS) that, unlike pre-post design, allowed controlling for pre-existing trends in utilization of maternal and newborn health services [19]. However, the current findings should still be interpreted with caution given potential study limitations. First, given that the survey was carried out from November 2014 to April 2015 and respondents were asked to report information that took place within five years before the survey, recall bias cannot be ruled out. This recall bias might have affected the pre-intervention and post intervention differentially and, thus, introducing differential misclassification bias on our outcome measures. However, this would bias us toward finding an impact and we found none. Similarly, we could not rule out the possibility of social desirability bias that could have affected the reporting health services use. Second, there are other relevant indicators (e.g., neonatal and maternal referrals to higher levels of care, and outcomes of care provided) that we could have examined that are not in RHDS. Lastly, our sample size was not large enough to allow us to perform a stratified analysis to study whether the impact of RapidSMS differed by the additional UNICEF support described earlier. However, we were able to generate reasonably stable monthly estimates to study the impact of RapidSMS nationwide.

In conclusion, based on our analysis using national representation survey data, we found that the implementation of RapidSMS did not increase uptake of the maternal and newborn health services we studied in Rwanda. In most instances this was the result of our indicators being very high, leaving little room for further improvement. Sustainable reduction of maternal and neonatal mortality in line with the SDG targets would certainly require increasing coverage of proven effective maternal and newborn interventions across the continuum of care. As such, exploring effective implementation strategies, including well designed mhealth interventions, that are likely to improve coverage of life-saving maternal and newborn interventions is warranted. Equally important is rigorous evaluation of such interventions to guide policy decisions including scale up over time.
